# Supplementary material for: Modeling glioblastoma heterogeneity as a dynamic network of cell states
Source: Mol Syst Biol. 2021 Sep 16;17(9):e10105. doi: 10.15252/msb.202010105 (PMC8444284; doi:10.15252/msb.202010105)
Supplement: Supplementary file 6 — Source Data for Figure 5 [file MSB-17-e10105-s004.zip › Figure5A_sourcedata/GSEA_3071/hallmarks_stateIV.GseaPreranked.1623742551702/HALLMARK_UV_RESPONSE_DN.html]

Details for gene set HALLMARK\_UV\_RESPONSE\_DN[GSEA]

|  || Dataset | state53071 |
| Phenotype | NoPhenotypeAvailable |
| Upregulated in class | na\_neg |
| GeneSet | HALLMARK\_UV\_RESPONSE\_DN |
| Enrichment Score (ES) | -0.4659568 |
| Normalized Enrichment Score (NES) | -2.3327208 |
| Nominal p-value | 0.0 |
| FDR q-value | 0.0 |
| FWER p-Value | 0.0 |
Table: GSEA Results Summary

  

Fig 1: Enrichment plot: HALLMARK\_UV\_RESPONSE\_DN      
 Profile of the Running ES Score & Positions of GeneSet Members on the Rank Ordered List

  

| PROBE | GENE SYMBOL | GENE\_TITLE | RANK IN GENE LIST | RANK METRIC SCORE | RUNNING ES | CORE ENRICHMENT || 1 | CELF2 |  |  | 63 | 1.766 | 0.0103 | No |
| 2 | MMP16 |  |  | 90 | 1.578 | 0.0254 | No |
| 3 | NFIB |  |  | 117 | 1.491 | 0.0393 | No |
| 4 | COL1A2 |  |  | 137 | 1.425 | 0.0538 | No |
| 5 | PLPP3 |  |  | 198 | 1.264 | 0.0583 | No |
| 6 | SRI |  |  | 298 | 1.037 | 0.0525 | No |
| 7 | INPP4B |  |  | 328 | 0.991 | 0.0595 | No |
| 8 | MAGI2 |  |  | 331 | 0.987 | 0.0716 | No |
| 9 | CDKN1B |  |  | 374 | 0.939 | 0.0755 | No |
| 10 | DLG1 |  |  | 520 | 0.798 | 0.0578 | No |
| 11 | SDC2 |  |  | 571 | 0.747 | 0.0577 | No |
| 12 | ARHGEF9 |  |  | 688 | 0.667 | 0.0439 | No |
| 13 | AMPH |  |  | 716 | 0.650 | 0.0470 | No |
| 14 | ATRX |  |  | 727 | 0.645 | 0.0532 | No |
| 15 | CDON |  |  | 764 | 0.622 | 0.0542 | No |
| 16 | PIK3R3 |  |  | 852 | 0.578 | 0.0449 | No |
| 17 | RBPMS |  |  | 881 | 0.569 | 0.0467 | No |
| 18 | ATXN1 |  |  | 985 | 0.528 | 0.0336 | No |
| 19 | MAP2K5 |  |  | 988 | 0.526 | 0.0399 | No |
| 20 | HAS2 |  |  | 1066 | 0.502 | 0.0315 | No |
| 21 | BMPR1A |  |  | 1074 | 0.501 | 0.0366 | No |
| 22 | NR1D2 |  |  | 1082 | 0.499 | 0.0415 | No |
| 23 | IGF1R |  |  | 1140 | 0.482 | 0.0367 | No |
| 24 | PRKCA |  |  | 1214 | 0.461 | 0.0286 | No |
| 25 | INSIG1 |  |  | 1317 | 0.436 | 0.0145 | No |
| 26 | CAP2 |  |  | 1410 | 0.414 | 0.0021 | No |
| 27 | AKT3 |  |  | 1417 | 0.412 | 0.0062 | No |
| 28 | FYN |  |  | 1483 | 0.394 | -0.0013 | No |
| 29 | PTEN |  |  | 1652 | 0.358 | -0.0290 | No |
| 30 | ATRN |  |  | 1758 | 0.337 | -0.0448 | No |
| 31 | VLDLR |  |  | 1866 | 0.319 | -0.0613 | No |
| 32 | NIPBL |  |  | 1997 | 0.297 | -0.0825 | No |
| 33 | PTPN21 |  |  | 2131 | 0.279 | -0.1045 | No |
| 34 | RXRA |  |  | 2286 | 0.258 | -0.1308 | No |
| 35 | PRKAR2B |  |  | 2297 | 0.256 | -0.1295 | No |
| 36 | ZMIZ1 |  |  | 2414 | -0.259 | -0.1484 | No |
| 37 | ITGB3 |  |  | 2905 | -0.319 | -0.2385 | No |
| 38 | TJP1 |  |  | 3113 | -0.348 | -0.2738 | No |
| 39 | FZD2 |  |  | 3199 | -0.360 | -0.2855 | No |
| 40 | IRS1 |  |  | 3584 | -0.424 | -0.3538 | No |
| 41 | SMAD7 |  |  | 3586 | -0.425 | -0.3486 | No |
| 42 | NOTCH2 |  |  | 3853 | -0.477 | -0.3936 | No |
| 43 | ID1 |  |  | 3973 | -0.504 | -0.4101 | No |
| 44 | NFKB1 |  |  | 3989 | -0.507 | -0.4065 | No |
| 45 | KCNMA1 |  |  | 4212 | -0.570 | -0.4419 | No |
| 46 | IGFBP5 |  |  | 4280 | -0.592 | -0.4473 | No |
| 47 | CITED2 |  |  | 4303 | -0.598 | -0.4439 | No |
| 48 | ATP2B1 |  |  | 4385 | -0.631 | -0.4514 | No |
| 49 | RND3 |  |  | 4402 | -0.635 | -0.4464 | No |
| 50 | SYNE1 |  |  | 4477 | -0.669 | -0.4521 | No |
| 51 | SYNJ2 |  |  | 4550 | -0.705 | -0.4570 | Yes |
| 52 | FHL2 |  |  | 4576 | -0.723 | -0.4526 | Yes |
| 53 | ADORA2B |  |  | 4590 | -0.729 | -0.4459 | Yes |
| 54 | ATP2B4 |  |  | 4601 | -0.734 | -0.4385 | Yes |
| 55 | NEK7 |  |  | 4604 | -0.734 | -0.4295 | Yes |
| 56 | PDLIM5 |  |  | 4612 | -0.739 | -0.4215 | Yes |
| 57 | SLC7A1 |  |  | 4614 | -0.740 | -0.4123 | Yes |
| 58 | LTBP1 |  |  | 4661 | -0.769 | -0.4113 | Yes |
| 59 | NR3C1 |  |  | 4699 | -0.796 | -0.4083 | Yes |
| 60 | ANXA4 |  |  | 4817 | -0.882 | -0.4196 | Yes |
| 61 | BDNF |  |  | 4818 | -0.882 | -0.4084 | Yes |
| 62 | MYC |  |  | 4883 | -0.947 | -0.4087 | Yes |
| 63 | LPAR1 |  |  | 4963 | -1.034 | -0.4107 | Yes |
| 64 | ANXA2 |  |  | 4997 | -1.087 | -0.4032 | Yes |
| 65 | DLC1 |  |  | 5057 | -1.204 | -0.3993 | Yes |
| 66 | DUSP1 |  |  | 5090 | -1.265 | -0.3893 | Yes |
| 67 | COL5A2 |  |  | 5097 | -1.282 | -0.3742 | Yes |
| 68 | MT1E |  |  | 5129 | -1.359 | -0.3629 | Yes |
| 69 | MET |  |  | 5153 | -1.438 | -0.3490 | Yes |
| 70 | SERPINE1 |  |  | 5162 | -1.501 | -0.3315 | Yes |
| 71 | COL11A1 |  |  | 5169 | -1.526 | -0.3133 | Yes |
| 72 | NRP1 |  |  | 5177 | -1.553 | -0.2949 | Yes |
| 73 | TFPI |  |  | 5212 | -1.705 | -0.2798 | Yes |
| 74 | EFEMP1 |  |  | 5226 | -1.830 | -0.2591 | Yes |
| 75 | F3 |  |  | 5230 | -1.856 | -0.2361 | Yes |
| 76 | SNAI2 |  |  | 5238 | -1.940 | -0.2128 | Yes |
| 77 | MGLL |  |  | 5239 | -1.942 | -0.1881 | Yes |
| 78 | DAB2 |  |  | 5250 | -2.114 | -0.1632 | Yes |
| 79 | PDGFRB |  |  | 5253 | -2.141 | -0.1364 | Yes |
| 80 | CAV1 |  |  | 5281 | -2.850 | -0.1054 | Yes |
| 81 | COL3A1 |  |  | 5287 | -3.508 | -0.0618 | Yes |
| 82 | COL1A1 |  |  | 5290 | -4.897 | 0.0000 | Yes |
Table: GSEA details [plain text format]

  

Fig 2: HALLMARK\_UV\_RESPONSE\_DN: Random ES distribution      
 Gene set null distribution of ES for **HALLMARK\_UV\_RESPONSE\_DN**
